# Shiga Toxin-Producing *Escherichia coli* (STEC) in Developing Countries: A 10-Year Review with Global Perspective

**DOI:** 10.3390/microorganisms13071529

**Published:** 2025-06-30

**Authors:** Ali Nemati, Ali Dadvar, Mark Eppinger, Zohreh Karimpour, Soroush Saberi Kakhki, Alireza Sabeti Moghaddam Sabzevar, Mahdi Askari Badouei, Federica Gigliucci, Luis Fernando dos Santos, Keiji Nakamura, Hooman Javidi, Maryam Hafiz

**Affiliations:** 1 Department of Pathobiology, Faculty of Veterinary Medicine, Ferdowsi University of Mashhad, Mashhad 9177948974, Iran; bac.fum@gmail.com (A.N.); zohrehkarimpour1@gmail.com (Z.K.); soroushsaberikakhki@gmail.com (S.S.K.); alirezasabeti79@gmail.com (A.S.M.S.); askari.m@um.ac.ir (M.A.B.); hoomanhd1@gmail.com (H.J.); maryam.hafiz69@yahoo.com (M.H.); 2 Department of Microbiology, Tumor and Cell Biology, Karolinska Institutet, SE-171 77 Stockholm, Sweden; ali.dadvar@ki.se; 3 Department of Molecular Microbiology & Immunology, University of Texas at San Antonio, San Antonio, TX 78249-0600, USA; 4 South Texas Center for Emerging Infectious Diseases (STCEID), San Antonio, TX 78249-0600, USA; 5 European Union Reference Laboratory (EURL) for *Escherichia coli* Including Shiga Toxin-Producing *E. coli* (STEC), Department of Food Safety, Nutrition and Veterinary Public Health, Istituto Superiore di Sanità, 00161 Rome, Italy; federica.gigliucci@iss.it; 6 Center of Bacteriology (National Reference Laboratory for STEC Infections and HUS), Instituto Adolfo Lutz, São Paulo 01246-000, SP, Brazil; luis.santos@ial.sp.gov.br; 7 Department of Bacteriology, Faculty of Medical Sciences, Kyushu University, Fukuoka 812-8582, Japan; nakamura.keiji.046@m.kyushu-u.ac.jp

**Keywords:** Shiga toxin-producing *Escherichia coli*, STEC, developing countries, serogroup, serotype, human, animal, food

## Abstract

In the past two decades, Shiga toxin-producing *Escherichia coli* (STEC) has been responsible for multiple large-scale outbreaks worldwide, affecting thousands of individuals. While surveillance systems in developed countries such as the United States, the United Kingdom, Europe, Australia, Japan, and Canada are well-established, data on STEC prevalence in developing nations remain sparse, partly due to the absence of well-structured molecular diagnostic networks or surveillance systems. This review analyzed 250 studies published between 2014 and 2024 across 39 developing countries in Africa, Asia, Latin America, and the Caribbean, yielding 8986 STEC isolates. Detailed serogroup and serotype data were available for 55.9% of these, with O111, O157, and O26 being most common in humans. In animals, O157:H7 was most frequent, while food isolates mirrored global trends with O157 and O111 dominance. Notably, O145, a serogroup frequently reported in the U.S. and Europe, was absent from the ‘’Top Seven’’ serogroups. Shiga toxin subtypes *stx1a* and *stx2a* were most prevalent in human cases. In animal isolates, *stx2e* was the most prevalent subtype, while *stx2c* was most commonly found in food samples. We recommend establishing reference laboratories in these regions to improve data quality, strengthen monitoring efforts, and reduce the burden of STEC infections globally.

## 1. Introduction

Shiga toxin-producing *Escherichia coli* (STEC) is a major cause of intestinal infections and foodborne outbreaks worldwide [1,2]. In the European Union, STEC is the third most common foodborne pathogen, contributing to an estimated 2.8 million cases of enteric diseases annually [3]. Domestic animals, especially ruminants, play a key role as reservoirs for STEC infections, with transmission occurring primarily through direct contact or consumption of contaminated food [4].

The pathogenicity of STEC is primarily attributed to the production of Shiga toxins (Stx), which are classified into two main types, Stx1 and Stx2, based on sequence similarity, antigenic differences, and key biochemical characteristics [5,6]. These toxins are encoded by the *stx1* and *stx2* genes. The *stx1* gene has four subtypes—*stx1a*, *stx1c*, *stx1d*, and *stx1e*—while the *stx2* gene is more diverse, encompassing currently 15 known subtypes, from *stx2a* to *stx2o* [7,8]. Studies have shown that strains producing *stx2*, particularly the *stx2a* subtype, are more commonly associated with severe diseases like hemolytic uremic syndrome (HUS) [7,8,9]. This has led to the identification of STEC pathotypes based on *stx* profiles, which can inform both clinical management and public health strategies. Although this approach is not yet officially integrated into clinical practice, enhancing the use of *stx* subtyping—especially in regions with a high burden of STEC infections—could improve risk assessment, guide treatment decisions, and strengthen outbreak response measures by allowing more accurate identification of high-virulence strains. Another critical virulence factor in STEC is intimin, a protein encoded by the *eae* gene, which facilitates the intimate attachment of STEC to intestinal epithelial cells [10]. Strains that are intimin-positive are often linked to serious conditions, including hemorrhagic colitis (HC) and HUS [11,12,13,14]. Over 20 intimins mediating the attachment to intestinal cells have currently been described [12,13,15,16,17,18]. The combination of *eae* subtype *γ1* and *stx2a* is more associated with severe disease [12,19]. It is also important to note that disease severity is multifactorial, influenced by host factors, microbiota, and environmental conditions, and cannot be inferred solely from the STEC strain characteristics [20,21,22,23,24,25,26,27]. STEC includes over 400 serotypes, but severe human illness is usually caused by a relative small subset of serogroups, including non-O157 STEC serogroups O26, O45, O103, O111, O121, O145, referred to as the “Big Six”, which along with the globally dominant O157:H7, form the “Top Seven” [15,28,29].

Although the significance of STEC is well documented in developed countries, the situation in developing nations remains largely unclear. Many of these countries lack comprehensive data on key serogroups and serotypes, making it difficult to track outbreaks and identify sources of infection. Contributing factors may include limited financial resources, a shortage of trained personnel, inadequate laboratory infrastructure, and challenges related to sample collection and transportation. This review aims to fill this gap by reporting the distribution and virulence traits of STEC strains isolated from various sources across developing countries for the first time. Furthermore, this review seeks to highlight the worldwide distribution and public health impact of major STEC serogroups and serotypes, emphasizing the common prevalence patterns of dominant strains in both developed and developing nations and the importance of strengthening detection, surveillance, and control strategies, especially in food safety.

## 2. Materials and Methods

**Figure 1:**
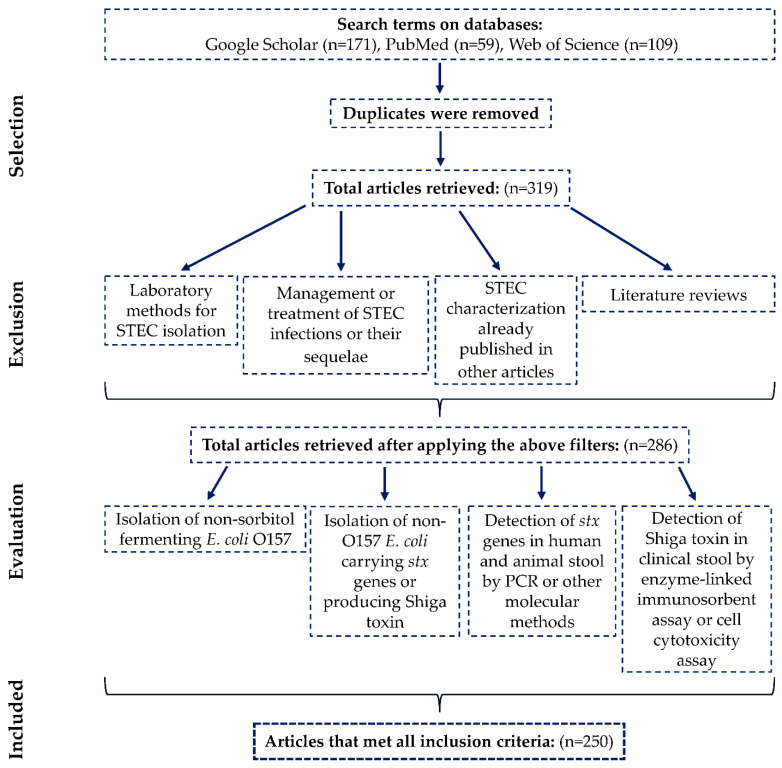
Flow chart of papers selection, exclusion, evaluation, and inclusion scheme.

Data for this review were collected from online databases including Google Scholar, PubMed, and Web of Science. We searched peer-reviewed literature, gray literature (e.g., preprints, surveillance data), and publicly available notifiable disease data (e.g., nationally reported, laboratory-confirmed STEC infections). The search was limited to studies published between January 2014 and October 2024, accessed between 19 February and 1 November 2024. We followed the Prisma guidelines (https://www.prisma-statement.org/, accessed on 5 September 2024), and our strategy for study selection is outlined in the provided flow diagram (Figure 1) [30,31]. The search terms used in these databases included Medical Subject Headings (MeSH) terms (https://www.nlm.nih.gov/mesh/meshhome.html, accessed on 19 April 2024): “Verotoxigenic”, “Verotoxigenic *Escherichia coli*”, “Verocytotoxin-Producing *Escherichia coli*”, “Verotoxin-Producing *Escherichia coli*”, “VTEC”, “Shiga Toxigenic *Escherichia coli*”, “Shiga Toxin-Producing *Escherichia coli*”, “STEC”, “Enterohemorrhagic *Escherichia coli*”, “EHEC”, along with the names of developing countries classified by the United Nations in the World Economic Situation and Prospects 2023 report (https://unctad.org/system/files/official-document/wesp2023_en.pdf, accessed on 1 January 2024). While VTEC and EHEC were valid search terms for finding relevant papers in this review, they are now generally considered obsolete or less precise in the scientific community. The more current and widely accepted terminology focuses on STEC as the preferred classification. Only articles published in English were included. Duplicates were removed, and a total of 319 papers were initially collected, regardless of sample size or detection methods. Each paper was then reviewed individually. Articles were excluded if they focused solely on laboratory methods for STEC isolation or diagnosis, microbiological characterization, the management and treatment of STEC infections or their sequelae, as well as strain characterizations already published in other articles (studies that used collection STEC strains, which had been previously analyzed), or were literature reviews. After applying these criteria, 286 papers were selected for evaluation (Figure 1).

The selected papers were evaluated based on the following criteria: isolation of non-sorbitol fermenting *E. coli* O157, isolation of non-O157 *E. coli* carrying *stx* genes or producing Shiga toxin, detection of *stx* genes in human and animal stool by polymerase chain reaction (PCR) or other molecular methods, and detection of Shiga toxin in clinical stool by enzyme-linked immunosorbent assay or cell cytotoxicity assay. Urinary and asymptomatic infections were excluded (Figure 1). The data were organized using Microsoft Excel 2019 (Microsoft Corporation), categorized by study DOI, year, country, serogroup, serotype, Shiga toxin-encoding genes (*stx1* and *stx2*), *stx* subtypes, and intimin-encoding gene (*eae*). The data were further grouped into three categories: humans, animals (cattle, sheep, goats, pigs, and pigeons), and foods (meat, milk, and vegetables).

## 3. Results

We reviewed 250 papers that provided data on STEC in 39 developing countries across Africa (17 countries), Asia (15 countries), and Latin America and the Caribbean (7 countries). These papers covered 11 regions, including East Africa (19 papers), West Africa (32 papers), North Africa (77 papers), Central Africa (1 paper), Southern Africa (9 papers), East Asia (28 papers), South Asia (46 papers), Western Asia (24 papers), the Caribbean (1 paper), Mexico and Central America (5 papers), and South America (8 papers). Information on STEC serogroups (O), serotypes (O:H), Shiga toxin genes (*stx1*, *stx2*) and respective subtypes, and the intimin gene (*eae*) was collected for 8986 cases, obtained from humans (1053 cases), animals (5292 cases), and foods (2641 cases). The animal isolates included cattle (2629), sheep (1047), goats (998), pigs (539), and pigeons (79). The food isolates came from meat (1386), milk (769), and vegetables (486).

### 3.1. Serogroups and Serotypes

**Figure 2:**
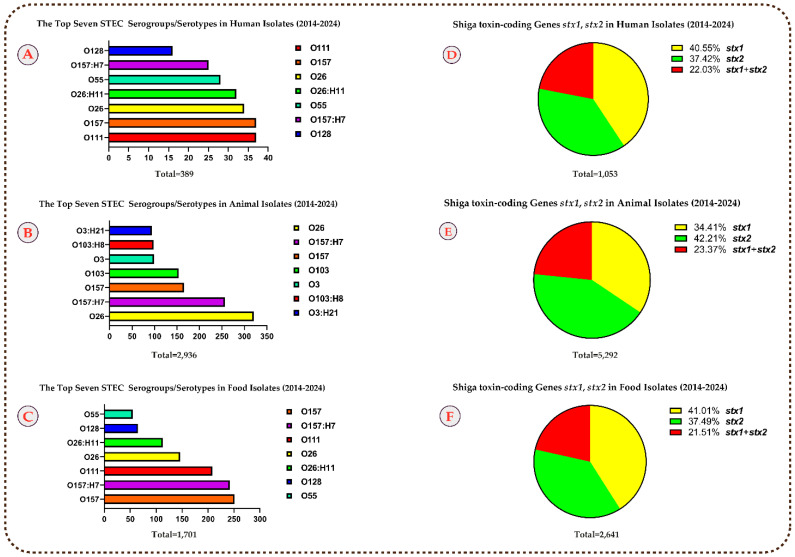
The ‘’Top Seven’’ most prevalent STEC serogroups/serotypes in humans (**A**), animals (**B**), and foods (**C**), and major Shiga toxin gene alleles in humans (**D**), animals (**E**), and foods (**F**), based on data collected in developing countries between 2014 and 2024.

Out of 8986 reported Shiga toxin-positive (*stx*-positive) isolates from developing countries between 2014 and 2024, data on STEC serogroups (O) and serotypes (O:H) were available for 5026 cases (55.9%) (Table 1 and Figure 2). These comprise 389, 2936, and 1701 isolates from humans, animals and food, respectively. Among the 389 human isolates, the 15 most frequently reported serogroups/serotypes were O111 (9.5%, 37/389), O157 (9.5%, 37/389), O26 (8.7%, 34/389), O26:H11 (8.2%, 32/389), O55 (7.1%, 28/389), O157:H7 (6.4%, 25/389), O128 (4.1%, 16/389), O111:H8 (3.8%, 15/389), O91:H14 (3.5%, 14/389), O104 (3.3%, 13/389), O91 (2.8%, 11/389), O145 (2.8%, 11/389), O128:H2 (2.5%, 10/389), O118 (1.7%, 7/389), and O151:H2 (1.7%, 7/389) (Table 1 and Figure 2).

Out of the 2936 reported animal isolates, the top 15 serogroups/serotypes were O26 (10.9%, 321/2936), O157:H7 (8.7%, 257/2936), O157 (5.6%, 166/2936), O103 (5.2%, 154/2936), O3 (3.3%, 99/2936), O103:H8 (3.3%, 98/2936), O3:H21 (3.2%, 94/2936), O45 (2.9%, 86/2936), O111 (2.7%, 82/2936), O43 (2.6%, 78/2936), O43:H2 (2.5%, 76/2936), O145 (2.1%, 64/2936), O8 (2.0%, 61/2936), O76:H19 (2.0%, 60/2936), and O76 (1.8%, 54/2936) (Table 1 and Figure 2).

For the 1701 food isolates, the 15 most detected serogroups/serotypes were O157 (14.7%, 251/1701), O157:H7 (14.2%, 242/1701), O111 (12.2%, 208/1701), O26 (8.5%, 146/1701), O26:H11 (6.6%, 113/1701), O128 (3.8%, 65/1701), O55 (3.2%, 55/1701), O103 (2.9%, 50/1701), O91 (2.8%, 48/1701), O121 (2.5%, 43/1701), O128:H2 (2.5%, 43/1701), O145 (2.4%, 41/1701), O45 (1.8%, 32/1701), O113:H21 (1.4%, 24/1701), and O146 (1.4%, 24/1701) (Table 1 and Figure 2).

### 3.2. Shiga Toxins and Intimin Genes

In 1053 isolates from humans, *stx1* was reported in 427 cases (40.5%), *stx2* in 394 cases (37.4%), and *stx1* and *stx2* in 232 cases (22.0%). Among 5292 animal isolates, *stx1* was detected in 1821 cases (34.4%), *stx2* in 2234 cases (42.2%), and both subtypes in 1237 cases (23.3%). In the case of 2641 STEC isolates from food sources, *stx1* was present in 1083 cases (41.0%), *stx2* in 990 cases (37.4%), and both *stx1* and *stx2* in 568 cases (21.5%). The intimin-coding gene *eae* was present in 43.3% of human cases (457/1053), 18.1% of animal isolates (959/5292), and 32.4% of food-sourced samples (858/2641) (Table 2 and Figure 2).

### 3.3. Stx Gene Subtypes

**Figure 3:**
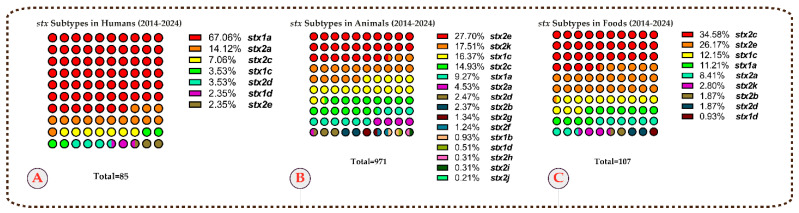
Prevalence of *stx* gene subtypes based on data reported for humans (**A**), animals (**B**), and foods (**C**) in developing countries from 2014 to 2024.

Among strains with reported subtyping data, the most common *stx* subtypes in human isolates were *stx1a* (67.0%, 57/85), *stx2a* (14.1%, 12/85), and *stx2c* (7.0%, 6/85). In animal isolates, the most prevalent *stx* subtypes were *stx2e* (27.7%, 269/971), *stx2k* (17.5%, 170/971), and *stx1c* (16.3%, 159/971). For food isolates, the most frequently detected *stx* subtypes were *stx2c* (34.5%, 37/107), *stx2e* (26.1%, 28/107), and *stx1c* (12.1%, 13/107) (Table 3 and Figure 3).

## 4. Discussion

Shiga toxin-producing *Escherichia coli* or STEC is a major cause of foodborne outbreaks worldwide [280,281]. It produces Shiga toxin, also known as verocytotoxin, which is structurally and functionally similar to the toxin produced by *Shigella dysenteriae* [7]. STEC has been historically categorized into different serogroups based on the O antigen in its cell wall and into serotypes based on both the O antigen and the flagellar H antigen [282]. This review provides the first comprehensive description of key serogroups/serotypes and Shiga toxin genes found in STEC isolates from human, animal, and food samples across developing countries, over a 10-year period (2014–2024).

**Figure 4:**
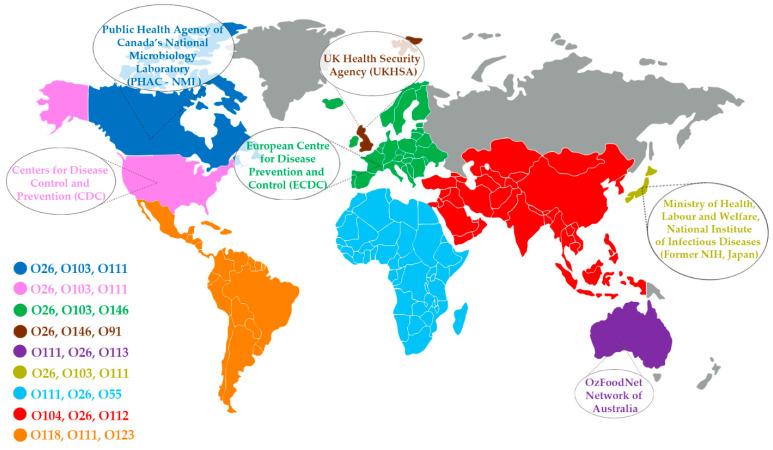
Global prevalence of the three most common non-O157 STEC serogroups. Countries with no available data in the surveyed databases are shown in gray (accessed October 2024).

The data reviewed here highlight the global distribution and public health significance of various STEC serogroups and serotypes, underscoring also a shared pattern of predominant STEC in both developed and developing countries (Figure 4). The data collected from developing countries we surveyed indicate that the serogroups O111, O157, and O26, along with the serotype O26:H11, are most frequently reported in human STEC cases, together accounting for 35.9% of all confirmed cases with known serogroups and serotypes. These serogroups are associated with severe human illnesses, including hemorrhagic colitis and HUS, and have been widely studied for their public health significance [29,283]. Serogroup O157, in particular, is a major cause of foodborne outbreaks, contributing to about 36% of all STEC infections in the United States, and has been linked to multiple large-scale outbreaks, including two involving ground beef in 2022 [284,285,286,287]. In Europe, O157 remains the most frequently detected serogroup, and in countries such as the United Kingdom, Australia, and Japan, human infections with O157 strains are consistently reported [3,288,289,290]. O111 is included in the USDA-FSIS’s list of “Big Six” non-O157 serogroups requiring monitoring in meat production and has also been implicated in clinical cases worldwide [291,292]. Similarly, O26:H11, another “Big Six” serotype, was responsible for a major outbreak in France in 2022, with 50 reported cases of HUS [3]. Moreover, O26 is the major non-O157 serogroup isolated from clinical samples reported in Japan and Canada [290,293]. As part of the USDA-FSIS “Big Six”, O26:H11′s prevalence in outbreaks in Europe reinforces its status as a high-risk pathogen. These trends illustrate the global significance of these serogroups in STEC infections, indicating a need for continued attention to their detection, monitoring, and control, especially in food safety practices.

The serogroups O26, O157, and O103, along with the serotype O157:H7, were the most frequently detected STEC strains in animal isolates across the regions surveyed. These findings highlight the presence of these serogroups with animal reservoirs, particularly cattle, where they persist in the intestinal tract and can contaminate meat during slaughter and processing [294]. The prevalence of O157:H7 in animals is particularly concerning due to its association with severe outbreaks worldwide, and as such, remains the most studied and monitored STEC lineage [1,5]. The “Big Six” serogroup O103 has also emerged as a significant pathogen. In Europe, it was ranked as the third most frequent STEC serogroup in human isolates between 2007 and 2022 [3]. In the United States, O103 is recognized as one of the most common non-O157 serogroups linked to foodborne illness [285]. In Japan and Canada, it is, after O26, the most frequently reported non-O157 STEC serogroup in clinical samples [290,293,295]. The frequent detection of these STEC strains in animal isolates underscores the importance of ongoing surveillance in livestock populations to better understand the dynamics of transmission from animals to humans via contaminated meat and dairy products.

In food isolates, O157, O157:H7, and O111 were most commonly detected, consistent with global trends [296]. These findings indicate that, similar to other parts of the world, the most commonly identified STEC strains in food sources in developing countries are those with a high pathogenic potential, such as O157 and O111.

Interestingly, O145, which is commonly reported in the U.S. and Europe, was notably absent from the ‘’Top Seven’’ serogroups in the developing countries surveyed. This discrepancy could be attributed to differences in detection capabilities, where less comprehensive or more targeted diagnostic methods in developing regions might focus on the better-known, high-risk serogroups [297]. Indeed, in developed countries, sequencing is also used, which allows the in silico determination of serogroup and serotype [298]. This advanced technology enables the detection of a broader range of serogroups, such as O145, that are not as readily identified in developing regions.

The ongoing global monitoring of these serogroups is crucial, as they are included in food safety guidelines such as the USDA-FSIS’s “Big Six”, and continue to be linked to outbreaks worldwide, reinforcing the need for heightened surveillance and control measures, especially in food production and processing environments. Figure 4 shows the three most frequently reported non-O157 STEC serogroups in different countries and regions, based on data accessed in October 2024 from public agencies and reviewed literature. The data cover both developed and developing nations, including Canada (O26, O103, O111), the United States (O26, O103, O111), Europe (O26, O103, O146), the United Kingdom (O26, O146, O91), Australia (O111, O26, O113), Japan (O26, O103, O111), Africa (O111, O26, O55), Asia (O104, O26, O112), and Latin America and the Caribbean (O118, O111, O123).

Among the human, animal, and food isolates, the highest rate of *eae*+ strains was observed in human samples (43.3%). The 2022 European surveillance report indicated that 44.8% of STEC strains isolated from severe human cases, including HUS and bloody diarrhea, were positive for both *stx2* and *eae*, further emphasizing the pathogenic potential of strains with these virulence traits in clinical settings [3,4]. In line with these findings, the survey also showed that *stx1a* and *stx2a* are carried by more than 81% of human isolates. In Europe, similarly, *stx2a* and *stx2d* were the most prevalent subtypes linked to severe human cases in 2022 [3]. *stx2*, particularly *stx2a* and *stx2d*, have been associated with elevated cytotoxicity and severe human illness, such as HUS [299,300,301,302] compared to the morbidity caused by *stx1a*+ STEC [303,304,305,306]. While *stx2* is often associated with more severe disease outcomes, the increased cytotoxicity of *stx1* in Vero cells, however, suggests that strains producing this toxin could also pose significant risks [300,307,308]. These findings underscore the critical role of these particular Shiga toxin suballeles in disease severity and clinical outcomes of STEC infections globally.

In animal isolates, *stx2e* was found to be the most prevalent Shiga toxin subtype. This observation might, however, be biased by the high number of *stx2e*-positive isolates reported from China, where *stx2e*-producing strains, primarily from pigs, have been frequently identified [169,170] (Table 3). While *stx2e* is clearly prevalent in animal populations [309], it has not been documented as a major culprit of human infections in Europe and the United States [3,7,283,285]. In food samples, *stx2c* was the most commonly reported Shiga toxin subtype in developing countries, and it was also the fourth most prevalent subtype of human infection in Europe in 2022, following *stx2a*, *stx1a*, and *stx2b* [3], which highlights the ongoing risk of foodborne transmission of *stx2c*+ strains.

Food products play a crucial role in the transmission via the ingestion of contaminated food, though they are not considered true reservoirs for STEC [4,5]. Overall, the high prevalence of *stx2a* in human infections and the increasing detection of *stx2c* in food isolates in developing countries emphasize the need for enhanced food safety measures and global surveillance efforts.

The data from our review highlight notable regional disparities in the reporting of STEC prevalence, with a clear gap observed in regions such as Central Africa, the Caribbean, Mexico, and Central America, where reports were less frequent compared to North Africa, South Asia, and West Africa (Table 4). This discrepancy may be linked to the economic conditions of low-income countries, which often face limitations in research funding and infrastructure, hindering comprehensive surveillance of STEC. Additionally, we observed regional variations in the major serogroups and serotypes reported (Table 4). These differences may be influenced by factors such as human-animal interactions (e.g., close contact with cattle, goats, and sheep), food production practices (e.g., traditional food preparation methods like raw milk consumption and fresh produce handling), and dietary habits (e.g., consuming raw or undercooked meat, seafood, and fresh vegetables) [310,311,312,313]. Recognizing these regional patterns is essential for developing targeted interventions to reduce the burden of STEC infections worldwide.

Among developing countries that reported STEC cases, only 55.9% included information on defined serogroups. This reflects limited surveillance capacity, with resources may be preferentially directed toward a small subset of well-known STEC serogroups, or a tendency to stop at identifying the STEC pathotype without further serogroup characterization, largely due to restricted diagnostic capabilities, particularly in molecular analyses and sequencing. Addressing these gaps is essential to improving STEC surveillance and strengthening our global understanding of its epidemiology.

## 5. Conclusions

Over the past two decades, STEC has been responsible for large-scale outbreaks, affecting thousands of people worldwide. Monitoring systems for STEC are well-established in countries like the United States, the United Kingdom, Europe, Australia, Japan, and Canada; however, there is a notable lack of data from developing countries, primarily due to the limited availability of organized molecular diagnostic networks and surveillance systems. The integration of genomics sequencing in STEC surveillance has significantly enhanced the ability to characterize STEC isolates [314,315]. However, many developing countries lack the infrastructure and resources for genomic-based and molecular biosurveillance. This review aims to fill that gap by providing an overview of STEC prevalence in regions with limited surveillance. To address this disparity, we recommend the establishment of reference laboratories for STEC in developing countries, similar to those operating in Argentina and Brazil. Such laboratories would enhance data quality, improve monitoring and tracking capabilities, and ultimately help reduce STEC infection. Furthermore, these reference laboratories could synergistically collaborate with existing laboratories, enabling the creation of more comprehensive global data on STEC prevalence in support of more effective public health strategies and interventions.

## Figures and Tables

**Table 1:** Data on the prevalence of Shiga toxin-producing *Escherichia coli* (STEC) serogroups (O) and serotypes (O:H) from humans, animals, and foods collected from online databases in developing countries from 2014 to 2024.

Source Country ^1,2^		Serotype ^3^		Year
	Humans	Animals ^4^	Foods ^5^	
Ethiopia	O157:ND (6) ^6^	O157:ND (72), O157:H7 (45)	O157:ND (8), O157:H7 (29)	2017–2023
South Sudan	O26:H11 (3), O111:H8 (2)	-	-	2023
Tanzania	-	O113:H21 (2), O157:H7 (4)	-	2014
Benin	-	O157:ND (2)	O157:ND (2)	2016–2020
Nigeria	O26:ND (2), O103:ND (1), O128:ND (1), O145:ND (1), O157:ND (10)	O26:ND (22), O91:ND (18), O103:ND (6), O111:ND (3), O157:ND (20), O157:H7 (8)	O26:ND (18), O45:ND (5), O91:ND (4), O103:ND (7), O111:ND (15), O121:ND (1), O128:ND (5), O138:ND (3), O145:ND (5), O157:ND (19), O157:H7 (11)	2014–2023
Senegal	-	O26:ND (2), O111:ND (1), O145:ND (1)	O111:ND (1)	2023
Algeria	-	O26:ND (8), O111:ND (5), O157:ND (2)	O26:ND (3), O111:ND (2), O157:ND (30)	2014–2020
Egypt	O3:ND (1), O6:ND (5), O26:ND (26), O26:H11 (14), O45:ND (5), O55:ND (28), O75:ND (1), O91:ND (10), O103:ND (2), O103:H2 (1), O104:ND (6), O111:ND (33), O111:H8 (6), O113:ND (2), O113:H4 (1), O117:ND (4), O121:ND (5), O128:ND (15), O128:H2 (10), O145:ND (6), O146:ND (1), O146:H21 (1), O157:ND (19), O157:H7 (11)	O6:ND (1), O26:ND (97), O26:H11 (18), O45:ND (45), O55:ND (7), O63:ND (1), O75:ND (3), O91:ND (13), O103:ND (12), O103:H2 (2), O111:ND (60), O113:ND (3), O113:H4 (1), O121:ND (14), O128:ND (23), O128:H2 (8), O145:ND (20), O145:H28 (3), O146:ND (3), O146:H21 (1), O157:ND (7), O157:H7 (9)	O26:ND (82), O26:H11 (111), O45:ND (3), O55:ND (53), O76:ND (1), O91:ND (37), O103:ND (13), O103:H2 (9), O104:ND (3), O111:ND (148), O111:H8 (12), O113:ND (3), O113:H4 (1), O113:H21 (1), O117:ND (16), O121:ND (31), O128:ND (52), O128:H2 (32), O145:ND (5), O146:ND (24), O146:H21 (10), O157:ND (54), O157:H7 (80), O166:H28 (21)	2014–2023
Sudan	O26:H11 (3), O111:H8 (2), O157:H7 (5)	-	-	2023
Tunisia	-	O45:ND (3), O103:ND (13), O145:ND (14), O157:H7 (10)	O26:ND (9), O45:ND (2), O91:ND (2), O103:ND (20), O121:ND (2), O145:ND (14), O157:ND (23)	2022–2024
Namibia	-	O103:ND (5), O145:ND (5), O157:H7 (10)	O26:ND (5), O45:ND (5), O103:ND (1), O111:ND (2), O121:ND (1), O145:ND (1), O157:ND (15)	2016–2020
South Africa	-	O2:ND (1), O3:ND (97), O3:H21 (94), O4:ND (1), O5:ND (3), O5:H19(3), O6:ND (15),O7:ND (1), O8:ND (42), O10:ND (3), O16:ND (1), O22:ND (3), O26:ND (157), O26:H11 (19),O43:ND (75), O43:H2 (73), O45:ND (37), O49:ND (1), O54:ND (2), O64:ND (1), O71:ND (3), O75:ND (37), O75:H8 (37), O76:ND (52), O76:H19 (50), O79:ND (1), O102:ND (1), O103:ND (101), O103:H2 (1), O103:H8 (98), O107:H7 (3), O108:ND (13), O111:ND (2), O111:H8 (8), O113:ND (8), O117:ND (7), O121:ND (10), O125:ND (1), O132:ND (1), O145:ND (23), O145:H8 (4), O146:ND (31), O146:H21 (31), O157:ND (44), O157:H7 (44), O159:ND (2), O162:ND (1), O163:ND (2), O168:ND (1), O174:ND (16), O175:ND (3), O176:ND (5), O185:ND (4)	-	2015–2022
China	O5:ND (2), O8:ND (1), O21:H25 (1), O26:H11 (5), O43:H2 (1), O84:ND (1), O91:ND (1), O112:ND (4), O157:H7 (2), O178:ND (1)	O2:ND (11), O5:ND (1), O5:H19 (4), O6:ND (1), O6:H10 (1), O8:ND (11), O8:H19 (4), O8:H21 (2), O8:H25 (5), O9:ND (4), O15:ND (1), O16:ND (8), O20:ND (24), O21:ND (1), O21:H25 (2), O22:H8 (12), O22:H16 (5), O44:ND (1), O48:H21 (1), O66:ND (2), O74:ND (2), O76:ND (1), O76:H19 (4), O81:ND (2), O84:ND (23), O86:ND (3), O87:ND (5), O93:ND (52), O100:ND (8), O100:H19 (3), O104:ND (1), O108:ND (4), O112:ND (1), O113:H4 (1), O114:ND (1), O116:ND (7), O128:H2 (1), O129:ND (1), O130:ND (1), O133:ND (11), O140:ND (1), O142:ND (1), O143:ND (3), O157:H7 (50), O159:ND (4), O172:ND (3), O174:ND (13), O177:ND (8), O184:H19 (17)	O5:ND (1), O5:H9 (1), O5:H19 (1), O8:ND (2), O8:H9 (3), O8:H19 (3), O8:H30 (2), O12:ND (2), O20:H21 (1), O21:H25 (2), O22:ND (2), O26:H11 (1), O40:H8 (2), O45:ND (5), O57:H21 (1), O76:H19 (2), O76:H21 (1), O84:ND (1), O91:ND (1), O91:H4 (4), O96:H19 (1), O98:ND (4), O100:H19 (3), O103:H25 (2), O104:H7 (3), O110:ND (2), O111:ND (1), O112:ND (2), O113:H4 (5), O113:H7 (1), O116:H21 (1), O118:ND (1), O120:ND (2), O121:ND (1), O128:H2 (10), O141:H29 (2), O145:ND (1), O150:ND (2), O157:ND (53), O157:H7 (76), O161:H19 (1), O172:ND (1), O174:ND (1), O176:H4 (5)	2014–2023
Malaysia	-	O130:H26 (1), O139:H1 (5), O157:H7 (32), O168:H21 (1)	-	2013–2015
South Korea	O26:H21 (1), O91:H14 (13), O103:H2 (1), O121:H19 (1)	O8:H19 (3), O8:H21 (1), O26:ND (3), O104:ND (1), O111:ND (1), O157:ND (5)	O1:ND (2), O6:ND (1), O18:ND (1), O18:H20 (1), O26:ND (1), O27:ND (1), O55:ND (2), O86:ND (1), O91:H14 (6), O91:H21 (2), O114:ND (1), O115:ND (1), O121:H10 (2), O125:ND (1), O126:ND (1), O128:ND (1), O157:H7 (4), O168:ND (1)	2014–2024
Thailand	-	O157:H7 (1)	-	2022
Bangladesh	-	O43:H2 (3), O57:ND (40), O76:H19 (6), O87:H16 (3), O110:H16 (1), O152:H8 (2)	O157:ND (7)	2015–2023
India	O28:ND (1), O59:ND (2), O64:ND (1), O69:ND (1), O85:ND (2), O95:ND (1), O102:ND (1), O103:ND (1), O157:H7 (1), O168:ND (1)	O2:ND (9), O3:ND (1), O4:ND (2), O5:ND (9), O7:ND (7), O8:ND (8), O9:ND (19), O10:ND (1), O11:ND (12), O12:ND (1), O17:ND (1), O20:ND (8), O21:ND (1), O22:ND (4), O24:ND (2), O25:ND (4), O26:ND (2), O27:ND (10), O30:ND (2), O33:ND (1), O34:ND (6), O41:ND (2), O43:ND (3), O52:ND (9), O55:ND (4), O56:ND (6), O59:ND (1), O60:ND (16), O63:ND (2), O69:ND (1), O74:ND (1), O75:ND (1), O76:ND (1), O81:ND (6), O83:ND (4), O84:ND (3), O85:ND (2), O89:ND (8), O91:ND (3), O95:ND (1), O97:ND (1), O100:ND (3), O103:ND (6), O110:ND (2), O112:ND (1), O113:ND (3), O116:ND (2), O118:ND (2), O119:ND (2), O121:ND (1), O123:ND (7), O125:ND (1), O134:ND (4), O136:ND (1), O137:ND (2), O138:ND (4), O147:ND (3), O152:ND (2), O156:ND (5), O157:ND (4), O165:ND (1), O168:ND (2), O173:ND (2)	O2:ND (1), O5:ND (2), O7:ND (7), O8:ND (6), O9:ND (1), O11:ND (1), O21:ND (1), O22:ND (2), O28:ND (1), O32:ND (1), O43:ND (4), O60:ND (6), O68:ND (5), O69:ND (2), O73:ND (3), O75:ND (1), O85:ND (2), O86:ND (4), O91:ND (1), O93:ND (1), O95:ND (1), O101:ND (1), O103:ND (1), O104:ND (1), O118:ND (1), O132:ND (1), O141:ND (1), O152:ND (2), O154:ND (2), O156:ND (2), O172:ND (1)	2014–2023
Iran	O26:ND (2), O45:ND (1), O121:ND (1), O157:ND (2), O157:H7 (1)	O5:ND (16), O5:H19 (3), O26:ND (29), O26:H11 (1), O26:H29 (1), O63:ND (1), O75:ND (3), O75:H2(3), O78:ND(3), O80:ND (2), O91:ND (3), O103:ND (11), O111:ND (10), O111:H8 (2), O113:ND (24), O113:H21 (3), O115:ND (2), O121:ND (1), O128:ND (10), O128:H2 (3), O145:ND (1), O157:ND (1)	O26:ND (25), O26:H11 (1), O45:ND (9), O91:ND (3), O103:ND (6), O111:ND (8), O113:ND (5), O121:ND (7), O121:H7(1), O128:ND (7), O128:H2 (1), O145:ND(5), O157:ND (34)	2014–2023
Pakistan	-	-	O9:H9 (2), O9:H30 (2), O82:H4 (3), O113:H16 (1), O187:H16 (1)	2017–2024
Iraq	-	-	O157:H7 (9)	2019–2023
Israel	O26:H11 (6), O71:H8 (1), O157:H7 (1)	O171:H21 (1), O171:H25 (1), O171:H29 (1)	-	2017
Saudi Arabia	-	-	O22:H8 (6), O111:ND (20), O113:H21 (12), O157:H7 (30)	2015
Turkey	O26:ND (4), O26:H6 (1), O26:H12 (1), O45:H2 (1), O103:H2 (2), O104:ND (7), O111:ND (1), O145:ND (3), O157:H7 (3), O174:H21 (2), O181:H4 (1)	O26:ND (1), O45:ND (1), O121:ND (22), O157:ND (9), O157:H7 (16)	O21:ND (2), O21:H25 (2), O45:ND (3), O103:ND (2), O111:ND (11), O145:ND (10), O157:ND (6), O157:H7 (3)	2016–2023
United Arab Emirates	-	O157:H7 (5)	-	2020
Costa Rica	O25:H4 (1), O71:H11 (2), O103:H2 (2), O111:H8 (3), O118:ND (7), O119:H2 (2), O137:H4 (1), O145:H28 (1), O151:H2 (7), O174:H21 (2)	-	-	2024
Mexico	-	O3:ND (1), O8:H7 (2), O8:H8 (2), O9:H19 (5), O15:ND (1), O39:H21 (1), O48:ND (3), O48:H8 (1), O48:H54 (1), O58:H40 (1), O73:ND (1), O75:ND (8), O77:H18 (1), O80:H39 (1), O88:H25 (10), O103:H25 (1), O104:ND (2), O112:H7 (1), O116:H16 (4), O116:H21 (3), O121:H11 (3), O146:H21 (7), O154:H1 (2), O154:H4 (2), O154:H32 (1), O157:H7 (17), O168:ND (1), O174:H28 (1), O175:H21 (3), O176:H11 (1), O176:H54 (1), O178:ND (1), O179:H8 (3), O180:ND (2)	-	2016–2023
Argentina	-	O1:H21 (1), O113:H21 (1), O130:ND (1), O130:H11 (1), O171:H2 (1), O174:H28 (1), O175:H19 (7), O178:H19 (5), O179:H8 (1), O187:H7 (1)	O8:H16 (4), O8:H19 (2), O22:H8 (3), O39:H49 (6), O91:H14 (1), O91:H40 (1), O113:H21 (8), O117:H7 (2), O130:H11 (5), O153:H28 (1), O160:H40 (1), O164:H8 (2), O171:H2 (2), O174:H21 (3), O174:H28 (2), O178:H19 (3), O185:H7 (8)	2017–2023
Brazil	O8:H19 (1), O24:H4 (1), O26:H11 (1), O71:H8 (1), O91:H14 (1), O100:ND (1), O103:ND (1), O111:ND (3), O111:H8 (2), O111:H11 (1), O118:H16 (1), O123:ND (3), O123:H2 (1), O145:ND (1), O153:H7 (1), O153:H21 (1), O157:H7 (1), O177:ND (1), O178:H19 (1)	O104:ND (2), O113:H2 (2), O157:H7 (5)	O8:H21 (1), O21:H19 (1), O22:H16 (1), O26:ND (3), O73:H45 (1), O79:H7 (3), O83:H19 (1), O113:H21 (3), O117:H7 (1), O132:H21 (1)	2018–2023
Uruguay	-	O8:H16 (2), O8:H19 (2), O20:H7 (1), O22:H8 (5), O74:H42 (1), O88:H25 (1), O99:H19 (1), O113:H21 (1), O116:H49 (1), O120:H7 (1), O156:H10 (2), O157:H7 (1), O159:H28 (1), O171:H2 (1), O174:H21 (2), O174:H28 (3), O178:H19 (2), O185:H25 (1)	-	2023

^1^ Countries were classified as developing based on the World Economic Situation and Prospects 2023 report by the United Nations (https://unctad.org/system/files/official-document/wesp2023_en.pdf, accessed on 1 January 2024). ^2^ Countries for which there were no data in the surveyed databases (PubMed, Google Scholar, Web of Science) were excluded from this table (accessed in October 2024). ^3^ ND: Not Defined. ^4^ Cattle, Sheep, Goats, Pigs, and Pigeons. ^5^ Meat, Milk, and Vegetables. ^6^ Number of serogroups and serotypes reported in each country.

**Table 2:** Data on the distribution of Shiga toxin-coding genes *stx1*, *stx2*, and intimin-coding gene *eae* in Shiga toxin-producing *Escherichia coli* (STEC) gathered from online databases covering humans, animals, and foods in developing countries from 2014 to 2024.

Source Country ^1^		Shiga toxin/Intimin		Year	Author
	Humans	Animals ^2^	Foods ^3^		
Ethiopia	*stx1* (29), *stx2* (28), *stx1*+*stx2* (30)/*eae* (42)	*stx1* (40), *stx2* (113), *stx1*+*stx2* (30)/*eae* (156)	*stx1* (3), *stx2* (15), *stx1*+*stx2* (5)/*eae* (13)	2017–2023	[32,33,34,35,36,37,38,39,40,41,42,43,44,45,46]
Kenya	*stx2* (6), *stx1*+*stx2* (13)/*eae* (9)	-	-	2018	[47]
South Sudan	*stx2* (5), *stx1*+*stx2* (5)	-	-	2023	[48]
Tanzania	-	*stx2* (5), *stx1*+*stx2* (2)/*eae* (4)	-	2014	[49]
Benin	-	*stx1* (2)/*eae* (2)	*stx1* (12), *stx2* (104)/*eae* (1)	2016–2020	[50,51]
Burkina Faso	-	*stx1* (2), *stx2* (2), *stx1*+*stx2* (1)/*eae* (1)	*stx2* (6)/*eae* (3)	2016–2017	[52,53]
Nigeria	*stx1* (101), *stx2* (88), *stx1*+*stx2* (71)/*eae* (78)	*stx1* (249), *stx2* (232), *stx1*+*stx2* (17)/*eae* (14)	*stx1* (257), *stx2* (166), *stx1*+*stx2* (69)/*eae* (139)	2014–2023	[54,55,56,57,58,59,60,61,62,63,64,65,66,67,68,69,70,71,72,73,74,75,76,77,78,79]
Senegal	-	*stx1*+*stx2* (7)/*eae* (8)	*stx1*+*stx2* (4)/*eae* (2)	2023	[80]
Algeria	-	*stx1* (168), *stx2* (22), *stx1*+*stx2* (7)/*eae* (68)	*stx1* (7), *stx2* (21), *stx1*+*stx2* (6)/*eae* (18)	2014–2020	[81,82,83,84,85,86]
Egypt	*stx1* (97), *stx2* (75), *stx1*+*stx2* (41)/*eae* (169)	*stx1* (45), *stx2* (63), *stx1*+*stx2* (52)/*eae* (93)	*stx1* (225), *stx2* (205), *stx1*+*stx2* (236)/*eae* (308)	2014–2023	[87,88,89,90,91,92,93,94,95,96,97,98,99,100,101,102,103,104,105,106,107,108,109,110,111,112,113,114,115,116,117,118,119,120,121,122,123,124,125,126,127,128,129,130,131,132,133,134,135,136,137,138,139,140,141,142,143,144,145,146,147,148,149,150,151,152,153,154,155]
Sudan	*stx2* (5), *stx1*+*stx2* (5)	-	-	2023	[48]
Tunisia	-	*stx1* (16), *stx2* (13), *stx1*+*stx2* (11), *eae* (8)	*stx1* (61), *stx2* (17), *stx1*+*stx2* (1), *eae* (28)	2022–2024	[156,157,158,159]
Cameroon	-	-	*stx1* (4), *stx2* (4), *stx1*+*stx2* (4), *eae* (6)	2017	[160]
Namibia	-	-	*stx1* (73), *stx2* (2), *stx1*+*stx2* (10), *eae* (84)	2016–2020	[61,161]
South Africa	-	*stx1* (223), *stx2* (384), *stx1*+*stx2* (380), *eae* (181)	-	2015–2022	[162,163,164,165,166]
Zambia	-	*stx1* (4), *stx2* (15), *stx1*+*stx2* (22)	-	2016	[167]
Zimbabwe	-	-	*stx2* (7), *eae* (1)	2020	[168]
China	*stx1* (6), *stx2* (3), *stx1*+*stx2* (5), *eae* (11)	*stx1* (215), *stx2* (485), *stx1*+*stx2* (97), *eae* (91)	*stx1* (47), *stx2* (165), *stx1*+*stx2* (85), *eae* (11)	2014–2023	[11,169,170,171,172,173,174,175,176,177,178,179,180,181,182,183]
Indonesia	-	*stx2* (1)	*stx1* (1)	2016	[184]
Malaysia	-	*stx1* (1), *stx2* (32), *stx1*+*stx2* (1), *eae* (32)	-	2013–2015	[185,186]
South Korea	*stx1* (14), *stx2* (20), *stx1*+*stx2* (22), *eae* (3)	*stx1* (17), *stx2* (87), *stx1*+*stx2* (52)	*stx1* (18), *stx2* (8), *stx1*+*stx2* (9), *eae* (3)	2014–2024	[187,188,189,190,191,192,193]
Thailand	-	*stx2* (1), *eae* (1)	-	2022	[194]
Vietnam	-	*stx2* (50)	-	2019	[195]
Bangladesh	-	*stx1* (25), *stx2* (58), *stx1*+*stx2* (15)/*eae* (6)	*stx1* (1), *stx2* (11)	2015–2023	[196,197,198,199,200,201,202]
India	*stx1* (26), *stx2* (12), *stx1*+*stx2* (9)/*eae* (5)	*stx1* (236), *stx2* (224), *stx1*+*stx2* (230)/*eae* (63)	*stx1* (101), *stx2* (88), *stx1*+*stx2* (71)/*eae* (78)	2014–2023	[203,204,205,206,207,208,209,210,211,212,213,214,215,216,217,218]
Iran	*stx1* (66), *stx2* (80), *stx1*+*stx2* (18)/*eae* (33)	*stx1* (115), *stx2* (132), *stx1*+*stx2* (50)/*eae* (127)	*stx1* (101), *stx2* (23), *stx1*+*stx2* (12)/*eae* (67)	2014–2024	[219,220,221,222,223,224,225,226,227,228,229,230,231,232,233,234,235,236,237,238]
Pakistan	-	-	*stx1* (144), *stx2* (13), *stx1*+*stx2* (25)/*eae* (9)	2017–2024	[239,240,241]
Iraq	*stx1* (13), *stx2* (18)/*eae* (33)	*stx1* (13), *stx2* (17), *stx1*+*stx2* (62)/*eae* (27)	*stx1* (12), *stx2* (15), *stx1*+*stx2* (2)/*eae* (6)	2019–2023	[242,243,244,245,246,247,248,249]
Israel	*stx2* (6), *stx1+stx2* (3)	*stx2* (2), *stx1+stx2* (1)	-	2017	[250]
Saudi Arabia	-	-	*stx1* (1), *stx2* (74), *stx1*+*stx2* (8)/*eae* (51)	2015	[251,252]
Turkey	*stx1* (10), *stx2* (19), *stx1*+*stx2* (4)/*eae* (14)	*stx1* (181), *stx2* (123), *stx1*+*stx2* (84)/*eae* (61)	*stx1* (13), *stx2* (14), *stx1*+*stx2* (13)/*eae* (29)	2016–2023	[253,254,255,256,257,258,259,260,261,262,263,264]
United Arab Emirates	-	*stx2* (12), *eae* (8)	-	2020	[265]
Trinidad and Tobago	-	*stx1* (9), *stx2* (10), *stx1*+*stx2* (4)/*eae* (1)	-	2016	[266]
Costa Rica	*stx1* (23), *stx2* (4), *stx1*+*stx2* (1)/*eae* (22)	-	-	2024	[267]
Guatemala	*stx1* (23), *stx2* (19)/*eae* (18)	-	-	2022	[268]
Mexico	-	*stx1* (42), *stx2* (46), *stx1*+*stx2* (25)/*eae* (4)	-	2016–2023	[269,270,271]
Argentina	-	*stx1* (5), *stx2* (50), *stx1*+*stx2* (17)/*eae* (1)	*stx1* (1), *stx2* (20), *stx1*+*stx2* (3)	2017–2023	[272,273,274]
Brazil	*stx1* (19), *stx2* (6), *stx1*+*stx2* (5)/*eae* (20)	*stx1* (207), *stx2* (38), *stx1*+*stx2* (54)	*stx1* (1), *stx2* (12), *stx1*+*stx2* (5)/*eae* (1)	2018–2023	[275,276,277,278]
Uruguay	-	*stx1* (6), *stx2* (17), *stx1*+*stx2* (16)/*eae* (2)	-	2023	[279]
Total	*stx1* (427), *stx2* (394), *stx1*+*stx2* (232)/*eae* (457)	*stx1* (1821), *stx2* (2234), *stx1*+*stx2* (1237)/*eae* (959)	*stx1* (1083), *stx2* (990), *stx1*+*stx2* (568)/*eae* (858)	2014–2024	-

^1^ Countries for which there were no data in the surveyed databases (PubMed, Google Scholar, Web of Science) were excluded from this table (accessed in October 2024). ^2^ Cattle, Sheep, Goats, Pigs, and Pigeons. ^3^ Meat, Milk, and Vegetables.

**Table 3:** Data on the frequency of *stx* gene subtypes in Shiga toxin-producing *Escherichia coli* (STEC) strains retrieved from online databases, focusing on humans, animals, and foods in developing countries from 2014 to 2024.

Source Country ^1^		Shiga toxin subtypes		Year
	Humans	Animals ^2^	Foods ^3^	
Ethiopia	*stx2a* (1), *stx2c* (3)	*stx2a* (6), *stx2c* (53)	*stx2a* (2), *stx2c* (4)	2017–2023
Tanzania	-	*stx2c* (4)	-	2014
Egypt	*stx1a* (1)	*stx2a* (4), *stx2b* (1), *stx2c* (4), *stx2d* (2), *stx2e* (1)	-	2014–2023
South Africa	-	*stx2c* (10), *stx2d* (10)	-	2015–2022
China	*stx1a* (3), *stx1c* (3), *stx2d* (1), *stx2e* (1)	*stx1a* (79), *stx1c* (133), *stx2a* (4), *stx2b* (13), *stx2c* (23), *stx2e* (216), *stx2g* (13), *stx2k* (170)	*stx1a* (12), *stx1c* (13), *stx1d* (1), *stx2a* (5), *stx2b* (2), *stx2c* (31), *stx2d* (1), *stx2e* (26), *stx2k* (3)	2014–2023
Malaysia	-	*stx1a* (1), *stx2c* (32)	-	2013–2015
South Korea	*stx1a* (2), *stx2a* (1)	-	*stx2c* (2), *stx2e* (2)	2014–2024
Vietnam	-	*stx2e* (50)	-	2019
Bangladesh	-	*stx2d* (1)	*stx2d* (1)	2015–2023
Iran	*stx1a* (6), *stx1d* (1)	-	-	2014–2024
Israel	*stx2a* (5)	*stx2c (1), stx2d (1)*	-	2017
Turkey	*stx1a* (4), *stx2a* (1)	*stx1a* (4), *stx2a* (2), *stx2b* (9), *stx2c* (11), *stx2d* (6), *stx2e* (2), *stx2f* (12), *stx2h* (3), *stx2i* (3), *stx2j* (2)	*stx2a* (2)	2016–2023
Costa Rica	*stx1a* (23), *stx2a* (4)	-	-	2024
Mexico	-	*stx1b* (9), *stx1c* (26), *stx1d* (1)	-	2016–2023
Argentina	-	*stx1a* (4), *stx2a* (24)	-	2017–2023
Brazil	*stx1a* (18), *stx1d* (1), *stx2c* (3), *stx2d* (2), *stx2e* (1)	-	-	2018–2023
Uruguay	-	*stx1a* (2), *stx1d* (4), *stx2a* (4), *stx2c* (7), *stx2d* (4)	-	2023
Total	*stx1a* (57), *stx1c* (3), *stx1d* (2), *stx2a* (12), *stx2c* (6), *stx2d* (3), *stx2e* (2)	*stx1a* (90), *stx1b* (9), *stx1c* (159), *stx1d* (5), *stx2a* (44), *stx2b* (23), *stx2c* (145), *stx2d* (24), *stx2e* (269), *stx2f* (12), *stx2g* (13), *stx2h* (3), *stx2i* (3), *stx2j* (2), *stx2k* (170)	*stx1a* (12), *stx1c* (13), *stx1d* (1), *stx2a* (9), *stx2b* (2), *stx2c* (37), *stx2d* (2), *stx2e* (28), *stx2k* (3)	2014–2024

^1^ Countries for which there were no data in the surveyed databases (PubMed, Google Scholar, Web of Science) were excluded from this table (accessed in October 2024). ^2^ Cattle, Sheep, Goats, Pigs, and Pigeons. ^3^ Meat, Milk, and Vegetables.

**Table 4:** The main reported serogroup (O), serotype (O:H), Shiga toxin (Stx), and *stx* gene subtype of humans, animals, and foods STEC strains in developing countries based on region, from 2014 to 2024.

Region ^1^		Serogroup, Serotype, Shiga Toxin		No. Papers ^4^
	Humans	Animals ^2^	Foods ^3^	
East Africa	O157, O26:H11, *stx1*+*stx2*, *stx2c*	O157, O157:H7, *stx2*, *stx2c*	O157, O157:H7, *stx2*, *stx2c*	19
West Africa	O157, *stx1*	O26, O157:H7, *stx1*	O157, O157:H7, *stx2*	32
North Africa	O111, O26:H11, *stx1*, *stx1a*	O26, O157:H7, *stx1*, *stx2a*, *stx2c*	O111, O26:H11, *stx1*	77
Central Africa	-	-	*stx1*, *stx2*, *stx1*+*stx2*	1
Southern Africa	-	O26, O103:H8, *stx1*+*stx2*, *stx2c*, *stx2d*	O157, *stx1*	9
East Asia	O112, O91:H14, *stx1*+*stx2*, *stx1a*	O93, O157:H7, *stx2*, *stx2e*	O157, O157:H7, *stx2*, *stx2c*	28
South Asia	O26, O59, O85, O157, O157:H7, *stx1*, *stx2*, *stx1a*	O57, O76:H19, *stx2*, *stx2d*	O157, O82:H4, *stx1*, *stx2d*	46
Western Asia	O104, O26:H11, *stx2*, *stx2a*	O121, O157:H7, *stx1*, *stx2c*, *stx2f*	O111, O157:H7, *stx2*, *stx2a*	24
Caribbean	-	*stx2*	-	1
Mexico and Central America	O118, O151:H2, *stx1*, *stx1a*	O75, O157:H7, *stx2*, *stx1c*	-	5
South America	O111, O123, O111:H8, *stx1*, *stx1a*	O104, O175:H19, O178:H19, *stx1*, *stx2a*	O26, O113:H21, *stx2*	8

^1^ Regions were classified by the United Nations (UN) in the World Economic Situation and Prospects 2023 report (https://unctad.org/system/files/official-document/wesp2023_en.pdf, accessed on 1 January 2024). ^2^ Cattle, Sheep, Goats, Pigs, and Pigeons. ^3^ Meat, Milk, and Vegetables. ^4^ Number of papers surveyed for each region.

## Data Availability

The original contributions presented in the study are included in the article, further inquiries can be directed to the corresponding author.
